# The Association between Attachment and Mental Health Symptoms among School-Going Adolescents in Northern Uganda: The Moderating Role of War-Related Trauma

**DOI:** 10.1371/journal.pone.0088494

**Published:** 2014-03-10

**Authors:** James Okello, Etheldreda Nakimuli-Mpungu, Seggane Musisi, Eric Broekaert, Ilse Derluyn

**Affiliations:** 1 Department of Psychiatry, Makerere University College of Health Sciences, Kampala, Uganda; 2 Department of Psychiatry, Gulu University, Gulu, Uganda; 3 Department of Orthopedagogics, Ghent University, Ghent, Belgium; 4 Department of Social Welfare Studies and Centre for Children in Vulnerable Situations, Ghent University, Ghent, Belgium; Max Planck Institute of Psychiatry, Germany

## Abstract

**Background:**

The association between attachment and mental health symptoms in adolescents in a post-conflict low resource setting has not been documented.

**Methods:**

We investigated the relationship between parent and peer attachment and posttraumatic stress, depression and anxiety symptoms in a sample of 551 adolescents aged 13–21 years old. Attachment quality was assessed using the Inventory of Parent and Peer Attachment (IPPA). Post-traumatic stress, depression and anxiety symptoms were assessed using the Impact of Events Scale Revised (IESR) and Hopkins Symptom Checklist for Adolescents (HSCL-37A) respectively. Gender differences in attachment relationships were determined using independent t-tests. Multivariate logistic regression was used to assess whether attachment relationships were independently associated with posttraumatic stress, depression and anxiety symptoms. Hierarchical linear regression analyses were conducted to explore the moderating role of war-related trauma.

**Results:**

Our analyses revealed gender differences in attachment to parents, with males reporting stronger attachment than females. Parental attachment was protective against depression and anxiety symptoms but not posttraumatic stress symptoms after adjusting for potential confounders. Alienation by parents was independently associated with an increase in these mental health symptoms while peer attachment was not associated with any of these symptoms. However, in situations of severe trauma, our analyses showed that peer attachment was significantly protective against post-traumatic stress symptoms.

**Conclusions:**

Secure parental attachment is associated with better psychosocial adjustment in adolescents affected by war. Further, adolescents with secure peer attachment relationships in situations of severe war trauma may be less likely to develop posttraumatic stress symptoms. Interventions to enhance peer support in this post conflict setting would benefit this vulnerable population.

## Introduction

There is worldwide recognition of adolescence as a period of rapid significant transformation in all aspects of functioning. Changes in psychosocial function include increasing reliance on peers for intimacy and social support and decreasing time spent with parents [1]. Nonetheless, several studies have shown that parental influence is important for psychosocial adjustment [2]. The absence of secure parental attachment has been associated with more engagement in high risk behaviors and more mental health symptoms in adolescence [3]. Much of the information regarding the association between attachment and mental health symptoms is provided by research studies from developed countries [2]–[7]. For example, secure attachment in adolescence appears to be a protective factor against the development of posttraumatic stress disorder (PTSD) in adolescents in three Nordic countries (Iceland, Finland and Faroe Islands) [6] and against the development of anxiety in Chinese university students [7]. In contrast, insecure attachment is associated with a greater level of mental health symptoms following negative life events [4]. These studies show that attachment research in adolescence is especially important in understanding the relationship between interpersonal trauma and psychopathology. The extent to which this information would apply in say, a post-conflict, low-resource country is unknown.

For example, the brutal civil war between the Ugandan Government forces and the Lord's Resistance Army (LRA) rebels over a twenty year period targeted children and adolescents who were forcefully abducted and forced to commit atrocities [8], [9]. In post-war northern Uganda, established outcomes among adolescents include most commonly PTSD, depression and anxiety [8]–[11] as well as risk behaviors [12]. A growing body of evidence has shown that interfamilial violence, poverty and war-related trauma have an additive effect on these outcomes [13], [14].

Early research focused primarily on the effect of war-related factors including internal displacement, abduction, death of loved ones and loss of property on mental health. Subsequent research has emphasized the importance of identifying explanatory mechanisms and protective factors, such as social support, resilience, coping and attachment style [15]–[18]. The linkages between war exposure and adjustment in adolescents are dependent in part on interpersonal factors such as attachment [19].

The extant research shows that females are more attached to their peers than males; whereas males are more attached to parents than females [20]. In keeping with attachment theory, interactions with inconsistent, unreliable, or insensitive attachment figures interfere with the development of a secure, stable mental foundation; reduce resilience in coping with stressful life events; and predispose a person to break down psychologically in times of crisis [21]. Although some studies found causal links in which psychological symptoms increase attachment insecurity, attachment insecurity can be viewed as a general vulnerability to mental disorders, with the particular symptoms depending on genetic, developmental, and environmental factors [22].

Stressful events have been found to moderate the relationship between attachment and mental health outcomes in a few empirical researches [2]. For example, findings from a recent study showed that stressful life events strengthened the association between attachment and mental health symptoms in adolescence [23]. Another study has shown that childhood adversities influence the association between insecure attachment and depression [24]. Luthar et al. suggested a more complex interactive process is useful to conceptualize protective and vulnerability processes [25]. However, the extent to which insecure attachment is associated with mental health symptoms in post-conflict low resource setting like northern Uganda is unclear.

In this paper, first, we explore gender differences in attachment relationships. Second, we assess whether attachment relationships were independently associated with post-traumatic stress, depression and anxiety symptoms. Lastly, we explore the moderating role of war-related trauma in the association between attachment and mental health symptoms. We hypothesize that 1) there would be gender differences in attachment relationships, 2) that secure attachment (defined as high scores of attachment) would be significantly protective against mental health symptoms and 3) stressful war events would moderate the association between attachment and mental health symptoms.

## Methods

### Ethics Statement

This study was approved by the Ethics Committee at the faculty of Psychology and Educational Sciences of Ghent, Gulu University Research Ethics Committee, and the Uganda National Council of Science and Technology. Permission and informed written consent was obtained from all head teachers of participating secondary schools. On the day of the interviews, research assistants explained the purpose of the study (which had been previously explained in writing to parents when we obtained written permission from each head teacher) to each head teacher and adolescents saying that the study was trying to understand adolescents' experiences during the war and their effects. Because of the difficulty of obtaining written informed consent from each parent, the approved procedure for obtaining consent was such that head teachers consented on behalf of adolescents whose parents did not return the written information leaflets, indicating no objection to the study and their child's participation. No parent returned the leaflets and no head teacher declined to consent. The IRBs specifically reviewed and approved this method of consent/assent. Study participants less than 18 years old provided oral assent and those 18 years old and above provided informed written consent. Those who declined or withdrew participation were not required to give reasons. Study participants were given pens as a gift for participation and opportunities for support and referral for mental health services were available to all participants.

### Study Setting and Population

Study participants were school-going adolescents recruited from seven out of fourteen government and private-operated secondary schools in Gulu district situated in Northern Uganda. Prior to 2006, the district had been the epicenter of over 20 years of armed conflict between the Ugandan Government and the LRA rebel group. Of the 14 schools in Gulu Municipality, we contacted seven schools, with additionally stratification by single/mixed-sex schools. The seven schools were selected for three reasons: 1) they had a representative number of private and public schools with a large number of enrollments, 2)students' characteristics were comparable to all students in the Gulu municipality area, and 3) the school head teachers were willing to participate in the study. All students from the second and third year of secondary school were included in the sampling frame, because we hoped to follow-up participants before they completed secondary education from their respective schools.

### Study Procedure

Study data were collected between August and September 2010. We compiled age-specific class lists in the selected schools and consecutively recruited 13–21 year olds. The eligibility criteria required participants to be in their second or third year of secondary education, aged between 13 and 21 years and had the ability to comprehend study procedures and provide informed consent/assent. Research assistants reviewed the study questionnaires with local mental health staff and teachers and pretested with non-participating students to ensure local validity. Research assistants distributed study questionnaires to students who were present in class on a given day and were eligible to participate in the study. All questionnaires were anonymous and self-administered in English, the official language used in all Ugandan schools. Participants completed their questionnaires in class during regular school hours, with the help of trained research assistants. The entire set of questionnaires took approximately an hour for the participants to complete. Finally, the convenience sample consisted of 600 students from the two classes, and 551 of them completed their questionnaires.

### Study Measures

#### Socio-demographic variables

A standardized socio-demographic questionnaire was used to obtain study participants' age, gender, ethnicity, living situation, marital status of parents and whether their parents were still alive or not.

#### Trauma exposure variables

Past childhood adverse events were assessed using the Adverse Childhood Exposure (ACE) questionnaire [26]. Selected ACE items were used to examine six context-specific intra-familial childhood adversities: not growing up with parents, death of a parent, financial adversity (i.e. dependence on food aid for at least six months), and physical abuse by parents and by other adults, sexual abuse, and witnessing fights between the parents before the age of 13 years. The Cronbach α for this sample was 0.94 indicating good reliability. A total ACE score was computed for each participant and was modeled as a continuous variable in our analyses.

War-related trauma was measured using a locally developed Stressful War Events (SWE) questionnaire. This 17-item questionnaire was designed specifically for war-affected adolescents in this region, and included questions referring to a specific war-related traumatic event, such as “Did you experience living in an internally displaced persons' camp?”

#### Mental health variables

Post-traumatic stress symptoms were assessed using the Impact of Event Scale–Revised (IES-R) [27], a 22-items scale consisting of three subscales of intrusion, avoidance and hyper-arousal. The IES-R has already been administered in war-affected adolescent populations in northern Uganda and Africa [28], [29]. Respondents were asked to identify a specific event endorsed on the SWE questionnaire as a reference point for completing the IES-R, and indicate how much it distressed them in the past month by rating each item on a 5-point Likert scale from 0 (not at all) to 4 (extremely). The Cronbach α for this sample was 0.94 indicating good reliability. A total IES-R score was computed for each participant and was modeled as a continuous variable in our analyses.

Depression and anxiety symptoms were assessed using the Hopkins Symptom Checklist-37 for Adolescents (HSCL-37A) [30], a self-report questionnaire that previously had been used in war-affected and refugee populations [29], [31]. Thirty-seven items inquire the severity of DSM-IV based symptoms associated with depression (15 items), anxiety (10) and externalizing problems (12), using a 4-point Likert scale from 1 (never) to 4 (always). The Cronbach's alphas for the depression and anxiety subscales were .83 and .78 respectively. Total depression and anxiety scores were computed for each participant and were modeled as continuous variables in our analyses.

#### Attachment to parents and peers

The quality of parental and peer attachment in adolescence was assessed using the Inventory of Parent and Peer Attachment [5]. The IPPA was developed in order to assess adolescents' perceptions of the positive and negative affective/cognitive dimension of relationships with their parents and close friends- particularly how well these figures serve as sources of psychological security. The instrument is a self-report questionnaire with a five point Likert-type scale response format whereby each item has five possible responses ranging from “almost never true” to “almost always true”. The revised version is comprised of 25 items in each of the mother, father, and peer sections, yielding three attachment scores. In each of these three sections, three broad dimensions are assessed: degree of mutual trust (sample item, “My mother/father/friend respects my feelings”); quality of communication (sample item, “When my mother/father/friend knows that something is bothering me, they ask me about it”); and extent of anger and alienation (sample item, “I don't get much attention from my mother/father/friends”).

For brevity, and consistent with Gullone et al., the response scale was simplified to a three-point Likert scale of 3(*almost always or always true*), 2(*sometimes true*) and 1(*almost never or never true*) [32]. The Cronbach α in this study for the mother, father, and peer sections was 0.85, 0.88 and 0.75 respectively. Total scores of each section and the three dimensions were computed for each study participant. When the total score for each participant was computed, the Alienation subscale items were reverse-scored. Parent and peer attachment were treated as continuous variables in all analyses conducted.

### Statistical Analyses

First, we performed simple logistic regression analyses to explore any gender differences in maternal, paternal and peer attachment relationships. Second, we conducted multivariate logistic regression analyses in which the association between attachment and mental health symptoms including depression, anxiety and posttraumatic stress symptoms was examined, adjusting for age, gender, orphan status and adverse childhood experiences. Third, to evaluate the moderating role of war-related trauma on the association between attachment and mental health symptoms, we conducted three separate hierarchical binomial logistic regression analyses for each outcome.

## Results

Of the 600 students approached to take part in the study, 551 (92%) completed the questionnaires. The mean age of the study participants was 16 .7(SD = 1.34); age range 13 years to 21 years. The mean number of traumatic events experienced was 6.5(SD = 3.8) with a range of 0 to 17. Detailed baseline characteristics of the study population are presented in Table 1.

**Table 1:** Socio-demographic characteristics of school-going war-affected adolescents in Northern Uganda.

Characteristic	N(%)
Age category(years)	
13–16	251(45.55)
≥16	300(54.45)
Ethnicity	
Acholi	492(89.29)
Other	59(10.71)
Religion	
Non-Christian	21(3.81)
Christian	530(96.19)
Orphan status	
Has both parents	284(51.54)
Lost one parent	182(33.03)
Lost both parents	85(15.43)
Living situation	
Lives alone	13(2.36)
Lives with siblings	61(11.07)
Lives with relatives	46(8.35)
Lives with parents	431(78.22)
Parental Marital Status	
Married	342(62.07)
Divorced or Separated	91(16.52)
Single	118(21.42)
Number of Trauma Events	
<3 trauma events	93(16.88)
3–6 trauma events	144(26.13)
>6 trauma events	314(56.99)

Using simple logistic regression models, we found that there were significant gender differences with regard to maternal and paternal attachment (Table 2). Males reported stronger parental attachment than females. Male adolescents were more likely to have trust in mothers and more communication with fathers than female adolescents. Trust in fathers and communication with mothers was comparable across gender. Alienation to parents was more in females than males. There were no gender differences in peer attachments.

**Table 2:** Gender differences in maternal, paternal and peer attachment relationships.

	Males		Females		Total (N = 551)		t
	Mean	SD	Mean	SD	Mean	SD	
Maternal							
Attachment security	36.29	7.00	33.65	8.94	35.01	8.10	3.74***
Trust	15.01	3.17	13.68	4.26	14.36	3.80	4.08***
Communication	13.18	3.03	12.69	3.61	12.94	3.34	1.62
Alienation	4.02	2.39	4.58	2.72	4.28	2.57	−2.37
Paternal							
Attachment security	32.74	9.01	29.89	9.99	31.40	9.59	3.31***
Trust	13.38	4.18	12.71	4.67	13.07	4.43	1.67
Communication	11.99	3.91	10.74	4.32	11.39	4.15	3.25**
Alienation	4.59	2.89	5.57	2.84	5.05	2.90	−3.58***
Peer							
Attachment security	33.76	6.08	33.98	6.69	33.86	6.37	−0.37
Trust	14.92	3.11	14.76	3.64	14.84	3.37	0.59
Communication	11.50	2.70	11.89	2.78	11.68	2.74	−1.55
Alienation	7.24	2.09	7.11	2.14	7.18	2.11	0.66

*p<0.5,

**p<.01,

***p<.001.

Table 3 presents the adjusted associations between attachment (parental and peer) and each of the three outcomes of depression, anxiety and posttraumatic stress symptoms. Both paternal and maternal attachment was protective against depression (β = 0.09, 95% CI = 0.14–−0.05, p<0.001 and β = 0.198, 95% CI = 0.27–−0.12, p<0.001 respectively) and anxiety symptoms (β = 0.05, 95% CI = 0.08–−0.02, p = 0.001 and β = 0.11, 95% CI = 0.16–−0.06, p<0.001 respectively) but not posttraumatic stress symptoms (β = 0.03, 95% CI = 0.15–−0.09, p = 0.66 and β = 0.02, 95% CI = 0.18–0.22, p = 0.86 respectively) after adjusting for potential confounders.

**Table 3:** Multivariate linear regression model: The association between attachment relationships and mental health symptoms adjusted for sex, age, orphan status and adverse childhood events.

Characteristics	Depression symptoms scores		Post-traumatic stress symptom scores		Anxiety symptoms scores	
	β Coefficient	SE	β Coefficient	SE	β Coefficient	SE
Maternal Att.	−0.198	0.04	0.017	0.105	−0.110	0.027***
Trust	−0.414	0.08***	−0.068	0.22	−0.232	0.06***
Communication	−0.3300	0.94***	0.349	0.26	−0.166	0.07**
Alienation	0.504	0.12***	0.215	0.34	0.268	0.09**
Paternal Att.	−0.094	0.02***	−0.027	0.06	−0.054	0.02***
Trust	−0.250	0.07***	0.245	0.19	−0.189	0.05***
Communication	−0.305	0.08***	−0.167	0.21	−0.253	0.05***
Alienation	0.504	0.11***	0.584	0.31*	0.302	0.08***
Peer Att.	−0.071	0.05*	0.134	0.12	−0.044	0.032
Trust	−0.169	0.09*	0.269	0.24	−0.072	0.06
Communication	−0.134	0.11	0.709	0.29**	−0.115	0.08
Alienation	−0.278	0.14	−0.410	0.37	−0.219	0.09

*p<0.05,

**p<0.01,

***p<0.001.

Abbreviations: Maternal att. Maternal attachment security, Paternal att. Paternal attachment security, Peer att. Peer attachment security.

Alienation by mothers was independently associated with an increase in depression (β = 0.50, 95% CI = 0.26–0.75, p<0.001) and anxiety (β = 0.26, 95% CI = 0.10–0.44, p = 0.002) but not post-traumatic stress symptoms (β = 0.22, 95% CI = −0.48–0.88, p = 0.52) while alienation by fathers was independently associated with an increase in depression (β = 0.50, 95% CI = 0.28–0.72, p<0.001) anxiety (β = 0.30, 95% CI = 0.15–0.46, p<0.001) and post-traumatic stress symptoms (β = 0.58, 95% CI = −0.02–1.18, p = 0.05). Alienation by peers was not associated with any of these symptoms. Interestingly, trustful peer relationships were protective against depression symptoms (β = −0.17, 95% CI = −0.34–0.003, p = 0.05) while communication with peers was associated with more posttraumatic stress symptoms (β = 0.73, 95% CI = 0.14–1.31, p<0.015).

**Figure 1:**
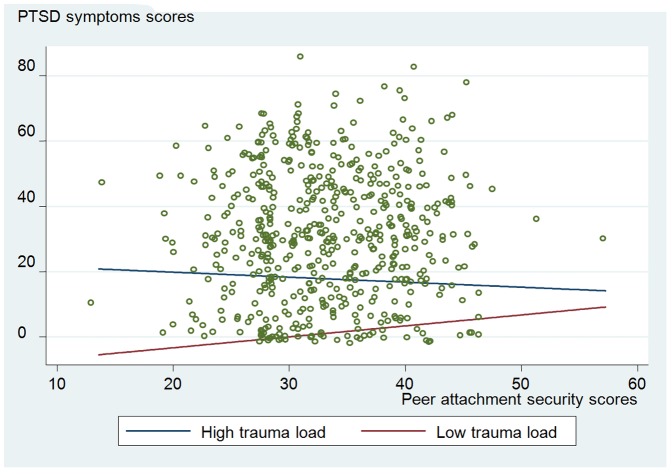
Interaction of trauma severity and peer attachment on post-traumatic stress symptoms. This figure reveals the moderation of trauma severity in the association between peer attachment and PTSD symptoms among war-affected adolescents. A significant interaction of trauma severity and peer attachment is found. Specifically, the relationship between peer attachment and PTSD symptoms is stronger among those with low levels of trauma than those with high levels of trauma. As peer attachment increases, participants with low levels of trauma will have more PTSD symptoms than those with high levels of trauma.

Table 4 presents the results of the moderation analyses. Fig. 1 shows that when there are high levels of stressful war events (1 standard deviation above the mean total number of stressful war events) increasing levels of peer attachment are associated with decreasing post-traumatic stress symptom scores (β = −0.17, 95% CI = −0.48–0.14, p = 0.28). On the other hand, when there are low levels of stressful war events (1 standard deviation below the mean total number of stressful war events) increasing levels of peer attachment are associated with increasing post-traumatic stress symptom scores (β = 0.32, 95% CI = 0.01–0.64, p = 0.043).

**Table 4:** Trauma severity moderates the association between peer attachment and post-traumatic symptoms.

Model	Step	Independent variable	β	95% CI	P-value
A	1	Peer attachment	0.131*	−0.12–0.38	0.297
	2	Peer attachment	0.08	0.14–0.32	0.442
		Traumatic events total scores	2.28	1.82–2.73	0.001
	3	Peer attachment	0.532	0.08–0.98	0.21
		Traumatic events total scores	4.5	1.12–6.98	0.000
		Peer attachment X traumatic events	–0.067	–0.12–−0.008	**0.026**

Abbreviations: CI, confidence interval.

*β –coefficient adjusted for age, sex, orphan status and adverse childhood events.

PTSD symptom scores were analyzed as a continuous variable.

Peer attachment and traumatic events total scores were also treated as continuous variables centered at the mean.

## Discussion

The present study aimed to examine the association between attachment and mental health symptoms and to explore the moderating role of war-related trauma in this relationship. We found three major findings. First, there were gender differences in attachment. Overall, males were more attached to their fathers and mothers than females, consistent with findings among non-trauma samples [20], [33]. Some researchers have reported that war trauma is especially more harmful to female than male relationships, possibly explaining the stability of male-parental attachment relationships [34].

Second, in keeping with our hypothesis, secure parental attachment was protective against both depression and anxiety symptoms. Contrary to the hypothesis, parental attachment was not associated with PTSD symptoms and peer attachment was not associated with any of the mental health symptoms. Specifically, the protective role of secure parental attachment against depression and anxiety symptoms is in keeping with what other studies, although executed in different contexts such as high-income and non-war-affected countries, have found [3], [7], [17].

It has been suggested that secure attachment, especially during adolescence may serve as a buffering system in the developmental stage of many internal and external pressures [6], [35],and that, according to a diathesis-stress model, one would therefore expect to find an interaction between number of stressful war events and level of attachment [3]. Regarding the association between parental attachment and post-traumatic stress symptoms, our findings are similar to what other researchers have found. Some studies have shown that secure parental attachment is not a significant contributor to posttraumatic stress [6], [36]. These researchers argue that adolescents receive greater social support following exposure to traumatic events and thereby manage to cope with the event in an adaptive way. Further, it has been observed that disclosure of painful traumatic experiences leads to resilience among traumatized children [37]. The inability to share painful traumatic experiences may therefore hinder the protective effects of parental attachment on PTSD symptoms.

Peer attachment was not protective against any mental health symptoms in our study sample. This finding is consistent with that of other researchers who have found a more robust protective effect of parental attachment than peer attachment against mental health symptoms [17], [38]. Some researchers have explained that the stronger protective role of parents compared to peers is due to the stronger family ties that overrule other relationships, especially during threat. Alternatively, they suggested that the atmosphere of suspicion surrounding war makes adolescents less trustful of disclosing experiences and sharing emotions with peers [34]. Feelings of alienation by parents, i.e. less secure attachment towards both parents, was associated with both depression and anxiety symptoms. It has been suggested that depressive cognitive schemata leads to feelings of rejection and distortion of the adolescent's feeling of being a person worthy of being loved [3].

Lastly, in contrast to our hypothesis, war-related trauma did not moderate the association between parental attachment and any of the mental health symptoms but it moderated the association between peer attachment and PTSD symptoms. Specifically, peer attachment was significantly protective against PTSD symptoms in situations of severe trauma. This finding contradicts previous studies in which severe trauma was reported to diminish positive peer relationships in which feelings of safety and togetherness are created [34]. A plausible explanation for this finding could be that the predominantly older adolescent population in this study would be more likely to seek social support and share emotions when facing traumatic events, which further can consolidate their friendships and protection from trauma-specific post-traumatic stress symptoms [33].

We note that war-related trauma did not moderate the relationship between parental attachment and mental health. Regardless of the level of war-related trauma, parental attachment remained protective against depression and anxiety symptoms. Possible explanations for this could be that the adolescents and their parents maintain strong attachment bonds irrespective of the severity of trauma, thus countering the effect of the stresses the adolescents may face. It is unclear why war-related trauma would significantly moderate the association between peer attachment and PTSD symptoms but not others.

These study findings should be viewed within the context of the following study limitations. First, all measures were based on retrospective self-report data, subject to recall and social desirability biases. Second, because we did not measure postwar stressors, we cannot rule out the influence of unmeasured aspects of the current psychosocial environment on the relation between attachment and mental health symptoms. Third, the sample was limited to school-going adolescents, affecting generalizability. Fourth, the study was cross-sectional, making it impossible to conclude a causal or temporal relationship between attachment and mental health symptoms. Fifth, our measure of attachment may not have captured certain aspects of the attachment relationship unique to the African ethnocultural setting, including whether the attachment indices were socially relevant, given that attachment as a concept is understudied in the African context [39].

Despite the above mentioned limitations, this study is the first to contribute to the scientific literature on attachment relationships and psychopathology in adolescents in a post-conflict low resource setting. Understanding the role of moderating influences is important for identifying individual differences in the influence of attachment on mental health and for targeting interventions that will hopefully improve mental health outcomes throughout the life span. Additional research is needed to develop more clearly the theoretical model concerning the relationships among war trauma, attachment, and psychosocial outcomes. In particular, there is a need to better understand the mechanism(s) through which attachment may serve as a protective factor against the negative effects of war-related trauma.

In conclusion, secure parental attachments are associated with better psychosocial adjustment in adolescents affected by war. Further, adolescents with secure peer attachment relationships in situations of severe war trauma may be less likely to develop posttraumatic stress symptoms. Interventions to enhance peer support in this post-conflict setting would benefit this vulnerable population.
